# Acute tumour response to a bispecific Ang-2-VEGF-A antibody: insights from multiparametric MRI and gene expression profiling

**DOI:** 10.1038/bjc.2016.236

**Published:** 2016-08-16

**Authors:** Lauren CJ Baker, Jessica KR Boult, Markus Thomas, Astrid Koehler, Tapan Nayak, Jean Tessier, Chia-Huey Ooi, Fabian Birzele, Anton Belousov, Magdalena Zajac, Carsten Horn, Clare LeFave, Simon P Robinson

**Affiliations:** 1 Cancer Research UK Cancer Imaging Centre, Division of Radiotherapy and Imaging, The Institute of Cancer Research, London SM2 5NG, UK; 2 Roche Pharma Research and Early Development (pRED), Roche Innovation Center, Penzberg DE-82377, Germany; 3 Roche pRED, Roche Innovation Center, Basel CH-4070, Switzerland; 4 Roche pRED, Roche Innovation Center, Welwyn AL7 1TW, UK; 5 Roche pRED, Roche Innovation Center, New York, NY 10016, USA

**Keywords:** antiangiogenesis, resistance, VEGF-A, Ang-2, MRI

## Abstract

**Background:**

To assess antivascular effects, and evaluate clinically translatable magnetic resonance imaging (MRI) biomarkers of tumour response *in vivo*, following treatment with vanucizumab, a bispecific human antibody against angiopoietin-2 (Ang-2) and vascular endothelial growth factor-A (VEGF-A).

**Methods:**

Colo205 colon cancer xenografts were imaged before and 5 days after treatment with a single 10 mg kg^−1^ dose of either vanucizumab, bevacizumab (anti-human VEGF-A), LC06 (anti-murine/human Ang-2) or omalizumab (anti-human IgE control). Volumetric response was assessed using T_2_-weighted MRI, and diffusion-weighted, dynamic contrast-enhanced (DCE) and susceptibility contrast MRI used to quantify tumour water diffusivity (apparent diffusion coefficient (ADC), × 10^6^ mm^2^ s^−1^), vascular perfusion/permeability (*K*^trans^, min^−1^) and fractional blood volume (fBV, %) respectively. Pathological correlates were sought, and preliminary gene expression profiling performed.

**Results:**

Treatment with vanucizumab, bevacizumab or LC06 induced a significant (*P*<0.01) cytolentic response compared with control. There was no significant change in tumour ADC in any treatment group. Uptake of Gd-DTPA was restricted to the tumour periphery in all post-treatment groups. A significant reduction in tumour *K*^trans^ (*P*<0.05) and fBV (*P*<0.01) was determined 5 days after treatment with vanucizumab only. This was associated with a significant (*P*<0.05) reduction in Hoechst 33342 uptake compared with control. Gene expression profiling identified 20 human genes exclusively regulated by vanucizumab, 6 of which are known to be involved in vasculogenesis and angiogenesis.

**Conclusions:**

Vanucizumab is a promising antitumour and antiangiogenic treatment, whose antivascular activity can be monitored using DCE and susceptibility contrast MRI. Differential gene expression in vanucizumab-treated tumours is regulated by the combined effect of Ang-2 and VEGF-A inhibition.

Angiogenesis, the development of new blood vessels to provide a nutritive blood supply, is a prerequisite for solid tumour growth and survival (Ferrara and Kerbel, 2005). Angiogenesis is stimulated and orchestrated by the release of specific growth factors from tumour cells, endothelial cells or associated macrophages, the production of which is upregulated by physiological conditions associated with tumours, such as hypoxia, and which are associated with a compromised and chaotic blood supply.

The binding of vascular endothelial growth factor-A (VEGF-A) to its endothelial receptors is a key driver of tumour progression, angiogenesis and vascular permeability. Vascular endothelial growth factor is considered the most potent angiogenic growth factor, whose expression is also associated with poor prognosis in a wide range of human cancers (Yancopoulos *et al*, 2000). Consequently, a number of antibody and small molecule-based approaches have been developed to inhibit VEGF signalling. Of these, the most notable is bevacizumab, a humanised monoclonal antibody to VEGF-A with marked clinical efficacy (Hurwitz *et al*, 2004). Evasive resistance mechanisms to anti-VEGF monotherapies are, however, proving a clinical challenge to maintain effective treatment (Bergers and Hanahan, 2008).

The contribution of the Tie-2 receptor ligands angiopoietin-1 (Ang-1) and angiopoietin-2 (Ang-2) to tumour vascular remodelling has also been established (Tait and Jones, 2004). Ang-1 is expressed by pericytes, smooth muscle cells and fibroblasts, and is thought to drive vessel stabilisation and maturation (Yancopoulos *et al*, 2000). Angiopoietin-2 is primarily secreted by endothelial cells and stored in Weibel–Palade bodies (Fiedler *et al*, 2004). In contrast to Ang-1, Ang-2 destabilises vessel assembly, increases vessel permeability and induces a state of vascular plasticity, promoting endothelial cell sprouting and proliferation (Holash *et al*, 1999; Maisonpierre *et al*, 1997; Yancopoulos *et al*, 2000). Angiopoietin-2 expression is upregulated in a broad range of human malignancies (Hu and Cheng, 2009). Strategies that block the Ang-2 pathway have demonstrated antitumour effects, providing direct evidence that therapeutic targeting of this pathway may be of clinical benefit (Oliner *et al*, 2004; Mazzieri *et al*, 2011; Thomas *et al*, 2013). Importantly, studies adopting a combination approach using agents targeted against both VEGF and Ang-2 signalling have revealed superior activity over targeting either pathway alone (Brown *et al*, 2010; Hashizume *et al*, 2010). The interception of multiple angiogenic pathways thus provides both a complimentary and additive antitumour response.

Vanucizumab (RO5520985 or RG7221, Roche, Penzberg, Germany), a novel heterodimeric bivalent bispecific human antibody against Ang-2 and VEGF-A (bevacizumab-based) has recently been described (Kienast *et al*, 2013). Preclinical studies with vanucizumab demonstrated broad efficacy in a wide variety of tumour models, resulting in tumour growth delay, regression and an enhancement in chemotherapeutic response. Antiangiogenic effects of vanucizumab revealed a modulation in the tumour vasculature, including a reduction in tumour vessel density, mature vessel architecture, interstitial fluid pressure and inhibition of early tumour cell dissemination (Kienast *et al*, 2013; Bessho *et al*, 2015).

Advances in imaging technologies such as magnetic resonance imaging (MRI) provide a means of defining noninvasive quantitative biomarkers to inform on biologically relevant structure–function relationships in tumours. This enables an understanding of the behaviour and heterogeneous distribution of such associations, and informs on response and resistance to treatment (O'Connor *et al*, 2008, 2014). For the evaluation of novel oncology therapeutics, the use of pharmacodynamic biomarkers, including those afforded by imaging techniques, is now considered mandatory (Waterton and Pylkkanen, 2012; O'Connor *et al*, 2016). In addition to quantifying any therapy-induced volumetric change *in vivo*, functional MRI methods can provide additional mechanistic insight (Tunariu *et al*, 2012). Imaging biomarkers for assessing tumour response require evaluation before they can be deployed in clinical trials. In particular, the evaluation of imaging–pathology correlation, and whether changes in the imaging biomarker reflect desired changes in the underlying pathology, is important to establish, but can often only meaningfully be studied in animal models.

Vascular endothelial growth factor inhibitors including bevacizumab have been shown to elicit a tumour antivascular response that is detectable by dynamic contrast-enhanced (DCE) MRI (O'Connor *et al*, 2009; Loveless *et al*, 2012). However, the most informative functional imaging biomarker(s) for evaluating treatment response to combined Ang-2/VEGF blockade have not been established. Here we have used a multiparametric functional MRI approach to investigate tumour response to single-dose administration of vanucizumab in the Colo205 colon cancer xenograft model. The Colo205 model was selected given its documented sensitivity to Ang-2 and VEGF inhibition (Kienast *et al*, 2013; Oliner *et al*, 2004). The effects of vanucizumab on vessel perfusion, hypoxia, necrosis, vessel density and vessel maturation were also evaluated using *ex vivo* histopathology. Differences in the sensitivity of functional MRI biomarkers for assessing tumour response to the dual targeting of Ang-2 and VEGF-A are revealed. Preliminary analyses of vascular-related tissue biomarkers associated with tumour response to vanucizumab, identified by gene expression profiling, are also described.

## Materials and methods

### Cell culture, tumour propagation and treatment

Colo205 human colorectal cancer cells (ATCC CCL222 sourced in 2011, Teddington, UK), were cultured in RPMI-1640 medium supplemented with 10% fetal bovine serum and 2 mM L-glutamine (Invitrogen, Paisley, UK) and maintained at 37 °C in a humidified incubator with an atmosphere of 95% air, 5% CO_2_. Cells were authenticated by short tandem repeat (STR) profiling by ATCC and were not passaged for >6 months in our laboratory.

All experiments were performed in accordance with the local ethical review panel, the UK Home Office Animals (Scientific Procedures) Act 1986, the United Kingdom National Cancer Research Institute guidelines for the welfare of animals in cancer research (Workman *et al*, 2010), and the ARRIVE guidelines (Kilkenny *et al*, 2010). Female NCr *nu/nu* mice (7–8 weeks old, Charles River, Margate, UK) were injected with 5 × 10^6^ Colo205 cells subcutaneously in the right flank. Tumours were selected for imaging when volumes reached ∼250 mm^3^, as assessed by callipers, using the formula for ellipsoid volume, (*L* × *W*^2^)/2, where *L* and *W* were the two largest dimensions of the ellipsoid. Immediately following pretreatment MRI (day 0), mice were randomised and administered with a single 10 mg kg^−1^ intraperitoneal dose of either vanucizumab (RO5520985, anti-human/murine Ang-2/anti-human VEGF-A, Roche), bevacizumab (Avastin, anti-human VEGF-A, Roche), LC06 (anti-murine/human Ang-2) (Thomas *et al*, 2013) or omalizumab (Xolair, anti-human IgE control, Roche) (Kienast *et al*, 2013). Magnetic resonance imaging was repeated 5 days after treatment. An additional cohort of animals was treated but not imaged to provide an independent set of tumour samples for gene expression profiling.

### MRI data acquisition

Magnetic resonance imaging was performed on a 7T horizontal bore Bruker microimaging system (Ettlingen, Germany) using a 3 cm birdcage volume coil. Anaesthesia was induced with an intraperitoneal injection of fentanyl citrate (0.315 mg ml^−1^) plus fluanisone (10 mg ml^−1^ (Hypnorm; Janssen Pharmaceutical Ltd, High Wycombe, UK)), midazolam (5 mg ml^−1^ (Hypnovel; Roche)) and sterile water (1 : 1 : 2). A lateral tail vein was cannulated with a 27G butterfly catheter (Hospira, Royal Leamington Spa, Warwickshire, UK) for remote administration of MRI contrast agents. Mice were positioned in the coil on a custom-built platform to isolate the tumour, and their core temperature was maintained at 37 °C with warm air blown through the magnet bore.

Multi-slice T_2_-weighted contiguous 1 mm thick axial images were first acquired for localisation and subsequent quantitation of the tumour volume. Diffusion-weighted (DW) images were then acquired from three 1 mm thick axial slices across the centre of the tumour using an echo-planar imaging (EPI) sequence (3 × 3 cm field of view, 128 × 128 matrix, T_R_=3000 ms, 5 b-values ranging from 40 to 700 s mm^−2^, 4 averages). Diffusion-weighted MRI exploits the random diffusion of water molecules to measure changes in tissue cellularity, quantified through the measurement of the apparent diffusion coefficient (ADC, × 10^6^ mm^2^ s^−1^) (Padhani *et al*, 2009). Diffusion-weighted MRI is being actively exploited as an early biomarker of tumour response to targeted therapies (Baker *et al*, 2012).

Dynamic contrast-enhanced MRI data were then acquired from the central axial slice using an inversion recovery (IR) true-FISP sequence with one baseline scan (3 × 3 cm field of view, 128 × 96 matrix, T_I_=25–1451 ms, 50 inversion times, T_R_=2.4 ms, T_E_=1.2 ms, 8 averages), and 60 dynamic scans (T_I_=109–924 ms, 8 inversion times, T_R_=2.4 ms, T_E_=1.2 ms, scan T_R_=10 s, 1 average, 60° flip angle, temporal resolution=20 s) acquired for 3 min before and 17 min after an i.v. injection of 0.1 mmol kg^−1^ of a clinically approved, low-molecular-weight contrast agent Gd-DTPA (Magnevist, Schering, Berlin, Germany) (Baker *et al*, 2012). Dynamic contrast-enhanced MRI measures contrast agent extravasation from the blood plasma compartment to the extravascular extracellular compartment, that is, vascular leakage, typically expressed by the volume transfer constant *K*^trans^ (min^−1^). Increasing contrast agent concentration in the extracellular leakage space is related to both tumour perfusion and permeability; an effective vascular targeting drug would however be expected to reduce *K*^*trans*^ (Leach *et al*, 2005).

Susceptibility contrast MRI, using ultrasmall superparamagnetic iron oxide (USPIO) particles as an intravascular MRI contrast agent, was performed on a separate cohort of Colo205-bearing animals. Tumours were imaged at baseline (day 0), and 5 days post treatment with a single i.p. dose of 10 mg kg^−1^ of vanucizumab (*n*=6) or omalizumab (*n*=7). Multigradient-recalled echo (MGRE) images (T_R_=200 ms, T_E_=6 ms, T_Espace_=4 ms, 16 averages) were acquired from 3 contiguous 1 mm thick axial slices through the tumour, using a 128 × 128 matrix over a 3 × 3 cm field of view. A second set of identical MGRE images were acquired 2 min after intravenous injection of 150 *μ*molFe kg^−1^ USPIO particles (P904, Guerbet, Villepinte, France). The USPIOs create magnetic susceptibility variations in the proximity of perfused blood vessels, increasing the transverse MRI relaxation rate R_2_* of water in the surrounding tissue. Their long intravascular half-life enables steady-state measurements of tumour R_2_* to be made that are used to determine fractional blood volume (fBV, %) (Robinson *et al*, 2007).

### MRI data analysis

Tumour volumes were determined using segmentation from regions of interest (ROIs) drawn on T_2_-weighted images for each tumour-containing slice, where clear delineation of tumour from surrounding tissue could be determined. Diffusion-weighted, DCE and susceptibility contrast MRI data were fitted on a pixel-by-pixel basis using in-house software (Imageview, developed in IDL, ITT Visual Information Systems, Boulder, CO, USA). Diffusion-weighted and MGRE images were fitted using a Bayesian maximum *a posteriori* approach, allowing estimates of the median ADC and USPIO-induced change in R_2_* (ΔR_2_*) to be calculated, respectively (Walker-Samuel *et al*, 2009, 2010). Fractional blood volume (%) was subsequently quantified incorporating the ΔR_2_* data, as previously described (Burrell *et al*, 2011). For DCE MRI analysis, IR true-FISP data were fitted using a similar approach, utilising the dual-relaxation sensitivity (T_1_ and T_2_) of the pulse sequence and incorporating the Tofts and Kermode pharmacokinetic model, providing estimates of *K*^trans^ (Boult *et al*, 2011). In addition, model-free analysis was used to derive the initial area under the gadolinium uptake curve from 0 to 60 s after injection of Gd-DTPA (IAUGC_60_, mM Gd min).

### Immunofluorescence and immunohistochemistry

The hypoxia marker pimonidazole (60 mg kg^−1^; Hypoxyprobe Inc., Burlington, VT, USA) (Raleigh *et al*, 1999) was administered intraperitoneally immediately before the post-treatment MRI session to allow for full bioreduction in hypoxic tumour regions. Following MRI, Hoechst 33342 (15 mg kg^−1^; Sigma-Aldrich, Poole, UK), a marker for perfused vessels, was injected via a lateral tail vein (Boult *et al*, 2011; Baker *et al*, 2012). After 1 min, tumours were rapidly excised and bisected parallel with the imaging plane, with half of the tumour snap frozen over liquid nitrogen for immuno/fluorescence microscopy and gene signature analysis, and the other half fixed in formalin (10% (v/v) neutral buffered formalin) for subsequent paraffin embedding for immunohistochemistry and haematoxylin and eosin (H&E) staining.

Fluorescence signals from Hoechst 33342 and reduced pimonidazole adducts bound with mouse monoclonal FITC-conjugated antibodies were detected on whole tumour 10 *μ*m thick frozen sections using a motorised scanning stage (Prior Scientific Instruments, Cambridge, UK) attached to a BX51 microscope (Olympus Optical, London, UK), driven by image analysis software (CellP, Soft Imaging System, Münster, Germany) as previously described (Boult *et al*, 2011). Sections were then counterstained with H&E, dehydrated and imaged again using the same stage coordinates under bright-field illumination. CD31 was detected on adjacent sections using rat anti-mouse CD31 antibodies (1 : 100; BD Biosciences, Oxford, UK) and Alexa 546-conjugated goat anti-rat IgG antibody (1 : 500; Invitrogen) (Boult *et al*, 2011).

Hand-drawn ROIs were identified on the H&E image to ensure only tumour was included, and used to quantify the percentage of Hoechst 33342 uptake (vessel perfusion) and pimonidazole adduct area (hypoxia) using CellP as previously described (Baker *et al*, 2012). Microvessel density (MVD) was calculated from the mean vessel number (CD31) counted in >5 randomly selected high-power fields (× 100) for each tumour.

*α*-smooth muscle actin (*α*-SMA) immunohistochemistry was performed on 5 *μ*m thick formalin-fixed, paraffin-embedded (FFPE) sections using mouse monoclonal anti-*α*-SMA antibodies (1 : 500; 1A4, Sigma-Aldrich) and an anti-mouse HRP-labelled polymer (Dako, Ely, UK). The percentage of *α*-SMA-positive stained area was determined by analysing ⩾5 randomly selected fields at high magnification (× 200) from each tumour section using a colour deconvolution program in ImageJ (NIH, Bethesda, MD, USA). An adjacent section was stained with H&E and imaged under bright-field illumination. The percentage of tumour necrosis was determined using CellP as previously described (Baker *et al*, 2012).

### Next-generation RNA sequencing

Three to six tumours each from two independent experiments with the above-mentioned treatment groups were collected (cohort 1 no MRI, cohort 2 DW and DCE MRI imaged cohorts). Total RNA was isolated from frozen xenograft tissues following standard procedures (Qiagen RNease mini kit, Manchester, UK). RNA integrity was assayed using the Agilent Bioanalyzer system (Agilent Technologies, Stockport, UK), quantitated (by Ribogreen or Nanodrop, Thermo Scientific, Wilmington, DE, USA), and 500 ng of polyadenylated RNA passing the QC criterion of RIN >7 was subjected to sequencing library generation using Illumina TruSeq RNA sample preparation kit v2 (Illumina, Cambridge, UK) according to the manufacturer's instructions without modification. Final cDNA libraries were analyzed for size distribution, quantitated by qPCR, and then normalised to 2 nM in preparation for sequencing. Libraries were clustered using Illumina TruSeq PE Cluster Kit v3. Finally, the DNA sequence was determined on the HiSeq2000 using sequencing-by-synthesis technology via the Illumina TruSeq SBS Kit. For each specimen, 2 × 50 bp paired-end sequences were yielded at an approximate coverage of 50 million paired-end reads.

### Processing of RNA sequencing data

All RNASeq samples passed quality control in terms of number of reads per sample and read quality. Short reads were aligned to the human and mouse transcriptome (based on Ensembl v60) using Bowtie2 (Langmead and Salzberg, 2012). Based on the alignment, reads mapping to both transcriptomes at the same time were identified and discarded from further analysis. On average, 70% of the reads mapped to either transcriptome, with 7% of the reads mapping to both organisms that were subsequently filtered out. Across all samples, ∼80% of the reads mapped to human, whereas 20% mapped to mouse, likely representing the quantity of human tumour cells compared to mouse stroma and immune cells. The reads per kilobase of transcript per million mapped reads (RPKM) were then computed for each gene as described by Mortazavi *et al* (2008) using the gene composite length, that is, the sum of the length of all non-overlapping exon groups as normalisation factor, using in-house tools.

### Statistical analysis

Statistical analysis of MRI and histological data was performed with GraphPad Prism 5 (GraphPad Software, La Jolla, CA, USA). The mean of median values were used for statistical analysis of all quantitative MRI data, apart from tumour volumes, where mean values were used. A two-way ANOVA with Bonferroni correction, nonparametric Wilcoxon matched pairs signed-rank test or one-way ANOVA with Dunnett's multiple comparison test were used where appropriate, with a *P-*value of <5% considered significant. The ANOVA was applied on log2-transformed RPKM values for vanucizumab, bevacizumab or LC06 treated against control samples in order to identify differentially regulated genes. Multiple comparison correction was implemented across contrast pairs. Implementation of ANOVA for gene expression data was via R function *aov()* (http://www.statmethods.net/stats/anova.html).

## Results

### Treatment with vanucizumab slows tumour growth in Colo205 xenografts

**Figure 1:**
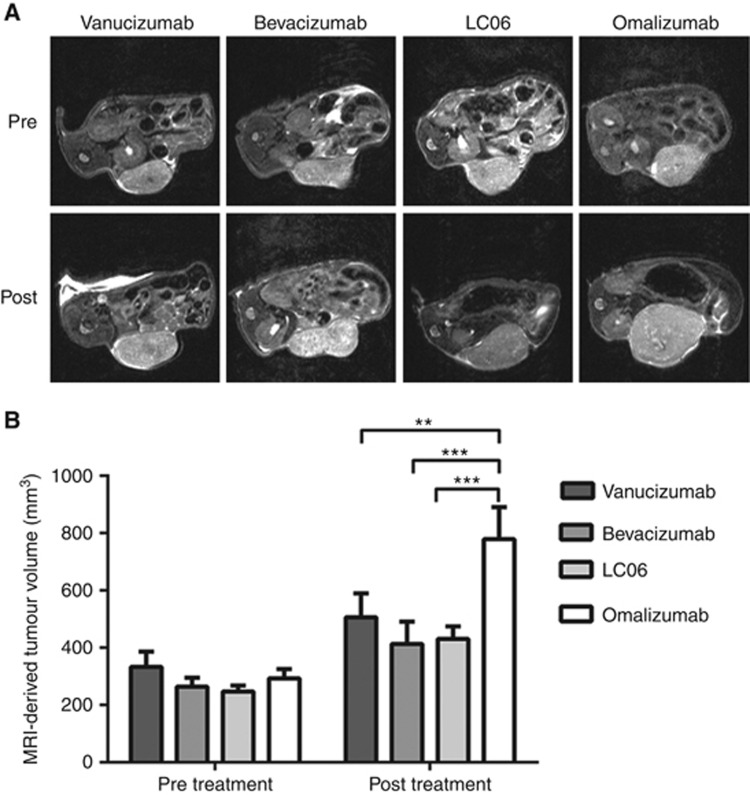
**Effects of vanucizumab on tumour progression.** (**A**) Transverse T_2_-weighted MR images of the central tumour slice acquired from paired mice bearing Colo205 xenografts before and 5 days after treatment, as indicated. (**B**) Summary of tumour volume response, determined by T_2_-weighted MRI, for each treatment cohort before and 5 days after treatment with vanucizumab, bevacizumab, LC06 or omalizumab (control). Data points are mean±1 s.e.m., *n*⩾6 per treatment, **P<0.01, ***P<0.001, two-way ANOVA with Bonferroni correction.

Tumour volumes were determined from multislice T_2_-weighted MR images before and 5 days post treatment with a single dose of vanucizumab, bevacizumab, LC06 or omalizumab. Representative T_2_-weighted images from all treatment groups are shown in Figure 1A. Treatment with vanucizumab resulted in a significant (*P*<0.01) cytolentic response (mean tumour volume increased by 52%) compared with control (166%). Treatment with either bevacizumab or LC06 induced a similar response, with significantly (*P*<0.001) lower mean tumour volumes compared with control (bevacizumab: 56% volume increase; LC06: 74%) (Figure 1B). No adverse treatment effects were observed in any cohort.

### Noninvasive assessment of tumour response to vanucizumab using DW and DCE MRI

Diffusion-weighted MRI was used to explore whether the antitumour effects of vanucizumab resulted in changes in ADC, an imaging biomarker associated with alterations in cell density and which typically increases with successful treatment. The mean ADC for each treatment group, at baseline and 5 days post treatment, are summarised in Table 1. No significant treatment-induced changes in mean tumour ADC were determined for any intervention, and there was no significant difference in the mean ADC of any treatment group after 5 days of treatment compared with omalizumab (control) (*P*>0.05).

**Figure 2:**
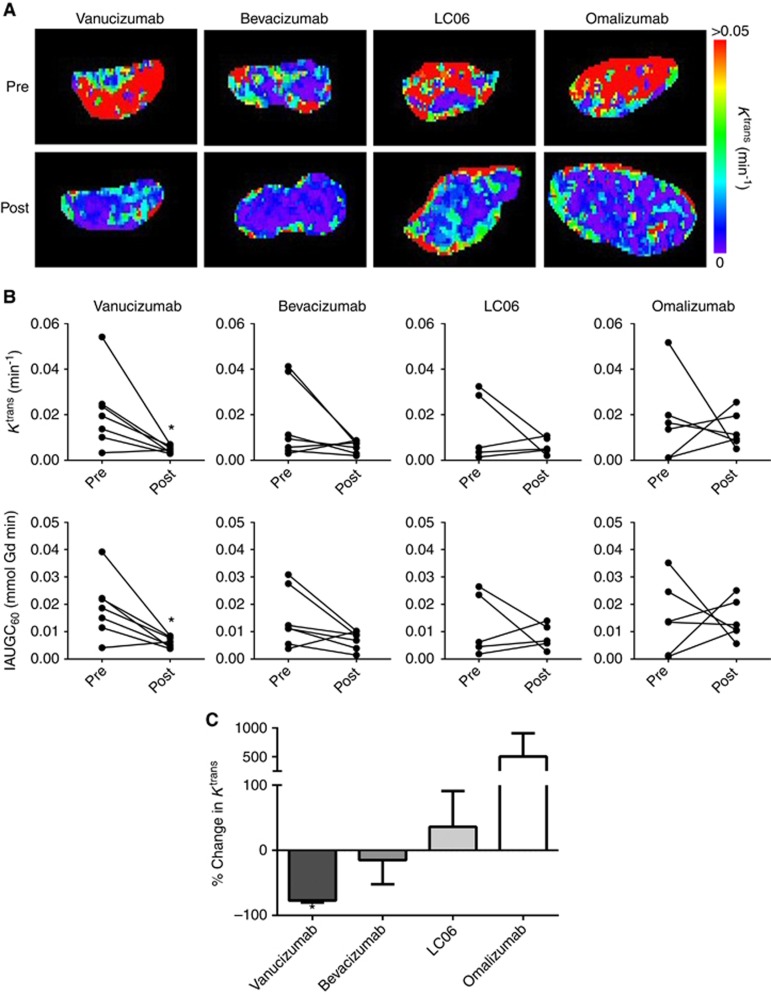
**Effects of vanucizumab on tumour vascular perfusion and permeability assessed by dynamic contrast-enhanced MRI.** (**A**) Parametric *K*^trans^ maps acquired from paired Colo205 xenografts before and 5 days after treatment with vanucizumab, bevacizumab, LC06 or omalizumab (control), as indicated. (**B**) Estimates of the transfer constant (*K*^trans^) and the initial area under the gadolinium uptake curve to 60 s (IAUGC_60_) determined from each Colo205 xenograft before and 5 days after treatment with vanucizumab, bevacizumab, LC06 or omalizumab. (**C**) Summary of the percentage change in *K*^trans^ determined across all four treatment groups. Data points are mean±1 s.e.m, *n*⩾6 per treatment, *P<0.05, Wilcoxon matched pairs signed-rank test. There was no significant difference in the change in *K*^trans^ 5 days post treatment with vanucizumab, bevacizumab or LC06 when compared with omalizumab (control) – two-way ANOVA with Bonferroni correction.

Dynamic contrast-enhanced MRI was used to assess any vascular response following treatment with vanucizumab, bevacizumab, LC06 or omalizumab. Representative parametric maps of *K*^trans^ are shown in Figure 2A. Colo205 tumours typically exhibited a marked, heterogeneous distribution of Gd-DTPA contrast agent uptake before therapy. After treatment, contrast agent uptake was noticeably restricted to the tumour periphery in all treatment groups. The quantitative data for *K*^trans^ and IAUGC_60_ is summarised in Figure 2B and C. A significant (*P*<0.05) reduction in both *K*^trans^ and IAUGC_60_ was determined in the vanucizumab-treated cohort only. There was however no significant difference in either DCE MRI biomarker 5 days post treatment with vanucizumab, bevacizumab or LC06 when compared with omalizumab (control).

### Decreased tumour perfusion following treatment with vanucizumab can be imaged using susceptibility contrast MRI

**Figure 3:**
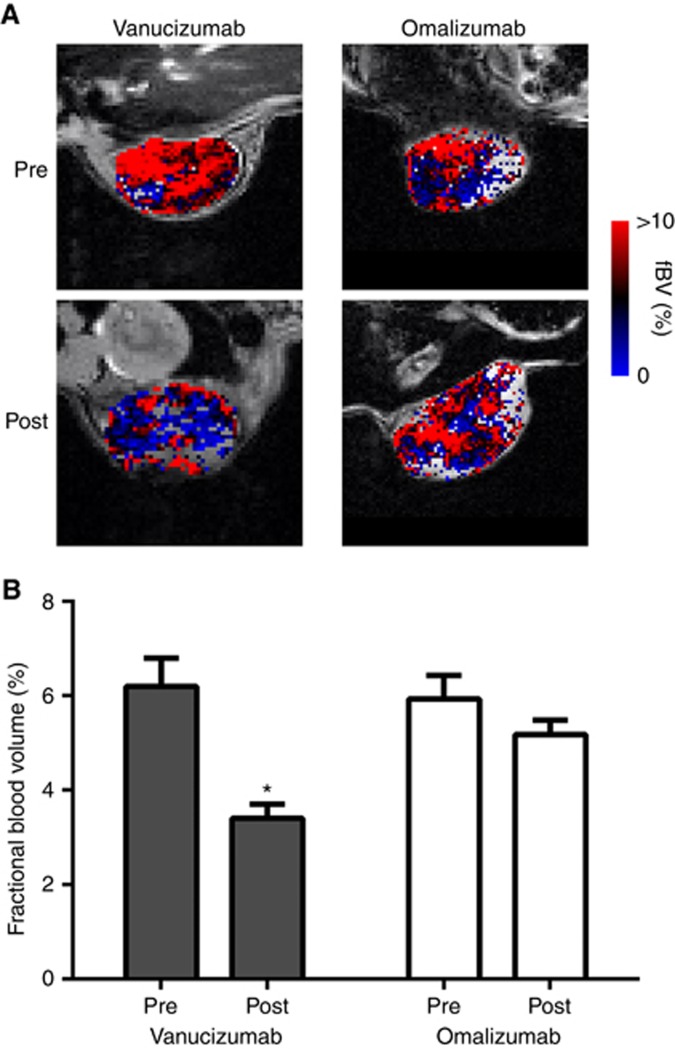
**Effects of vanucizumab on tumour fractional blood volume assessed by susceptibility contrast MRI.** (**A**) Parametric fBV maps acquired from the central slice of paired Colo205 xenografts before and 5 days after treatment with either vanucizumab or omalizumab (control) as indicated. (**B**) Summary of treatment-induced changes in tumour fBV, determined by susceptibility contrast MRI. Data points are mean±1 s.e.m., *n*⩾6 per treatment, *P<0.03, Wilcoxon matched pairs signed-rank test.

Representative parametric fBV maps acquired before and 5 days post treatment with either vanucizumab or omalizumab are shown in Figure 3A. The mean baseline fBV for all the subcutaneous Colo205 xenografts imaged was 6.1±0.4%. A marked reduction in USPIO particle delivery was evident in all the tumours following treatment with vanucizumab, equating to a significant (*P*<0.01) reduction in mean tumour fBV after 5 days of treatment (Figure 3B). The reduction in fBV 5 days post treatment was significantly (*P*<0.05) greater in the vanucizumab cohort (−2.7±0.5%) compared with that determined in the omalizumab cohort (−0.7±0.6%).

### Histological assessment of the effects of vanucizumab on perfused vasculature, hypoxia, microvessel density, vessel maturation and necrosis

**Figure 4:**
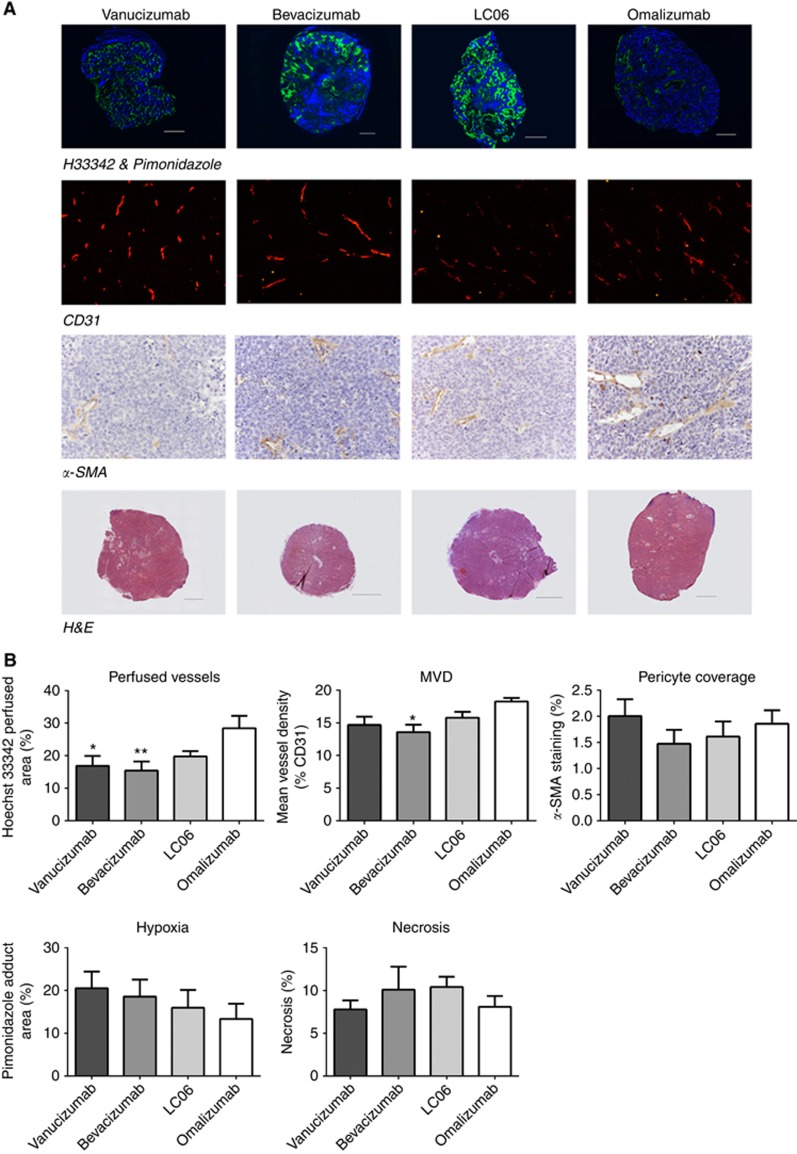
**Histological assessment of the effects of vanucizumab on tumour vasculature, hypoxia and necrosis.** (**A**) Composite fluorescence images of Hoechst 33342 uptake (blue, perfusion) and pimonidazole adduct formation (green, hypoxia) acquired from whole sections of Colo205 xenografts 5 days after treatment with vanucizumab, bevacizumab, LC06 or omalizumab (control). Fluorescence images (× 100 magnification) of CD31 immunohistochemistry used to assess tumour microvessel density (MVD) post treatment. High-magnification (× 200) images of *α*-SMA immunohistochemical staining used to assess pericyte coverage. Composite images of whole tumour sections stained with H&E used to assess necrosis. (**B**) Summary of treatment-induced changes in perfused tumour vessels, hypoxia, MVD, vascular maturation and necrosis. Data points are mean±1 s.e.m., *n*⩾5 tumours per treatment, *P<0.05, **P<0.01, one-way ANOVA with Dunnett's multiple comparison test.

Representative images acquired of each histological marker assessed for all treatment groups and control tumours are shown in Figure 4A, with the associated quantitative summary shown in Figure 4B. Composite fluorescence images revealed a heterogeneous distribution and negligible overlap of Hoechst 33342 uptake and pimonidazole adduct formation. Treatment with vanucizumab or bevacizumab resulted in significantly (*P*<0.05) lower tumour perfusion relative to control (vanucizumab: 17±3% bevacizumab: 15±3% control: 28±4%). There was no significant difference in hypoxia in any of the treatment groups compared with control. A single dose of either vanucizumab or LC06 had no significant effect on MVD 5 days after treatment compared with control (vanucizumab: 15±1% LC06: 16±1% control: 18±1%). In contrast, treatment with bevacizumab resulted in significantly (*P*<0.05) lower mean MVD compared with control (bevacizumab: 14±1%). Analysis of pericyte coverage revealed no significant difference in the expression of *α*-SMA following a single dose of vanucizumab, bevacizumab or LC06 compared with control. Haematoxylin and eosin staining revealed small localised regions of tumour necrosis (<20%) in all treated and control tumours. There was no significant difference in the mean percentage necrosis at 5 days post treatment in any treatment group compared with control.

### VEGF-dependent gene expression profiling

**Figure 5:**
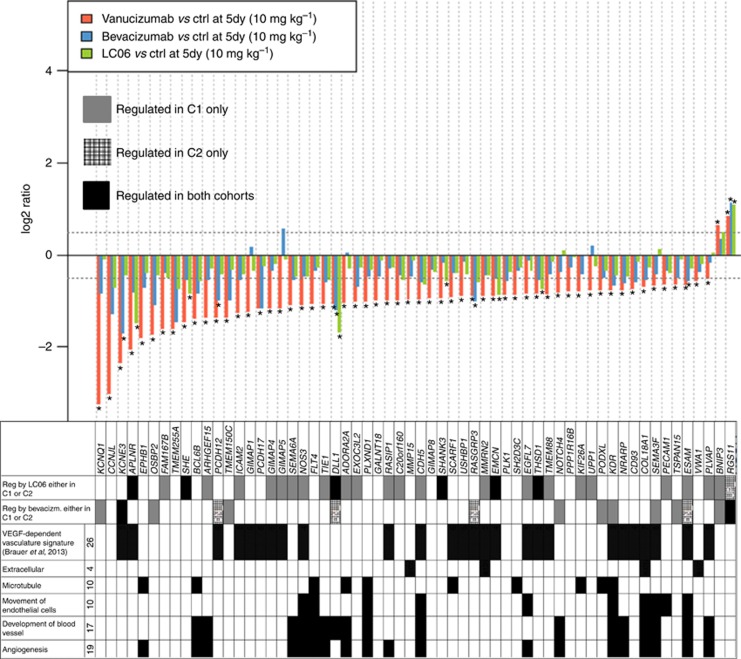
**Differential gene expression in vanucizumab-treated tumours.** Comparison of vanucizumab-treated tumours with omalizumab (control (ctrl)) tumour samples 5 days (5dy) after treatment led to the identification of 60 significant (*P<0.05 and |log_2_ ratio|>0.5) differentially expressed genes in the tumour stroma. The upper part of the Table shows a comparison with the respective results in the other treatment groups, whereas the colour code specifies if the gene was identified from cohort 1 (C1), cohort 2 (C2) or both. A total of 26 of all genes were previously described in the VEGF-dependent vasculature signature (Brauer *et al*, 2013). Where known, the particular biological process in which a gene is known to be involved with is indicated.

A total of 60 mouse Ensembl genes that could be mapped to human gene symbols were identified as significantly differentially expressed in the tumour when vanucizumab-treated tumour samples were compared with those treated with omalizumab 5 days after treatment and using the cutoff criteria of *P*<0.05 and |log_2_ ratio|>0.5 (Figure 5). Of these, the expression of 20 genes was altered only in the vanucizumab-treated tumours (Supplementary Figure S1a), 25 were altered by vanucizumab and LC06 only (Supplementary Figure S1b), 11 altered by vanucizumab and bevacizumab only and 4 were altered by all 3 antibodies relative to control. Where known, the biological function of the identified genes is indicated in Figure 5 and Supplementary Figure S1, and whether they had previously been described as VEGF-A-dependent genes by Brauer *et al* (2013).

## Discussion

Targeting tumour angiogenesis is an attractive treatment strategy, as the majority of solid tumours are dependent on a functional vascular supply, destruction of a single blood vessel results in the death of many tumour cells, and drug delivery issues are reduced as target cells are adjacent to the bloodstream. Recent studies suggest that adopting a multitargeted approach against several angiogenic pathways confers a therapeutic advantage over single-agent monotargeted therapy (Brown *et al*, 2010; Hashizume *et al*, 2010). One such strategy currently being investigated is the use of vanucizumab, a heterodimeric bivalent bispecific human antibody against Ang-2 and VEGF-A (Kienast *et al*, 2013). The antitumour efficacy of vanucizumab has been established in a panel of subcutaneous, orthotopic and patient-derived xenograft tumour models (Kienast *et al*, 2013; Bessho *et al*, 2015). However, as with any novel agent, accurate preclinical evaluation of the pathophysiological changes that occur *in situ* is essential for both the interpretation of therapeutic effects and in guiding clinical translation. In this regard, noninvasive MRI affords a range of quantitative imaging biomarkers that inform on tumour vascular architecture and function, and are often influenced by therapies targeted against tumour blood vessels (Tunariu *et al*, 2012). The aim of the present study was to investigate tumour response to a single dose of vanucizumab *in vivo* using a multiparametric MRI approach, validated *ex vivo* using histological methods.

Here we report that single-dose treatment with vanucizumab is sufficient to induce significant growth delay in the Colo205 xenograft tumour model as early as 5 days post treatment. Our results confirm that dual targeting of Ang-2 and VEGF-A provides similar growth inhibition to that achieved by targeting each pathway alone. We were unable to observe superior efficacy as has been previously reported following chronic treatment with vanucizumab in larger, more established Colo205 xenografts (Kienast *et al*, 2013).

Quantitation of tumour ADC using DW MRI is being actively investigated to detect changes in cellularity in response to treatment (Padhani *et al*, 2009). Typically, tumour ADC increases following successful therapy, and is associated with a loss in membrane integrity, an early feature of cellular death and increased necrosis. For example, single-dose treatment with bevacizumab has been shown to increase ADC in an orthotopic breast cancer model after 3 days (Moestue *et al*, 2013). Here, no significant difference in the ADC of Colo205 xenografts was determined following treatment with vanucizumab, bevacizumab or LC06. This imaging response correlates with the absence of any differences in pathologically determined tumour necrosis across all the treatment groups. These results are also in broad agreement with previous reports of no significant differences in cell apoptosis, necrosis or proliferative index following chronic treatment with vanucizumab (Kienast *et al*, 2013).

Dynamic contrast-enhanced MRI is the most common functional application of MRI in clinical oncology. Numerous studies have shown that, following intravenous injection of a low-molecular-weight gadolinium-chelated contrast agent, malignant tumours demonstrate faster and higher levels of signal enhancement than normal surrounding tissues, a consequence of their typically hyperpermeable vasculature, and this has underpinned the large clinical success of DCE MRI in tumour detection and diagnosis. Dynamic contrast-enhanced MRI has also been extensively used preclinically and clinically to assess tumour response to antiangiogenic and vascular disrupting agents (Leach *et al*, 2005; O'Connor *et al*, 2007). Thus, DCE MRI should be amenable to monitoring dual blockade of Ang-2 and VEGF by vanucizumab, previously shown to reduce tumour vascular density (Kienast *et al*, 2013). In the present study, a single 10 mg kg^−1^ dose of vanucizumab resulted in a significant reduction in both *K*^trans^ and IAUGC_60_ in Colo205 xenografts 5 days after treatment. No similar reduction in DCE MRI biomarkers was determined following treatment with bevacizumab or LC06. Importantly, vanucizumab also elicited a significant reduction in perfused vessels as measured by Hoechst 33342 uptake. Encouragingly, in the first-in-human phase 1 trial of vanucizumab, which incorporated DCE MRI, marked reductions in *K*^trans^ were observed as early as 96 h after initiation of treatment (Hidalgo *et al*, 2014).

Dynamic contrast-enhanced MRI-derived estimates of vascular function such as *K*^trans^ and IAUGC_60_, using a low-molecular-weight contrast agent, represent compound biomarkers of both tumour perfusion and permeability (Leach *et al*, 2005). Dynamic contrast-enhanced MRI measurements of tumour response to acute and chronic anti-VEGF treatment have been varied. Although several studies have reported a clear decrease in *K*^trans^ consistent with an antiangiogenic effect (see, e.g., Bradley *et al*, 2009; O'Connor *et al*, 2009), others have reported either no change following treatment (Desar *et al*, 2011) or a transient and prolonged increase in *K*^trans^, attributed to vascular normalisation of surviving vessels (Batchelor *et al*, 2007; Moestue *et al*, 2013). Antiangiogenic therapy may prune VEGF-dependent neoangiogenic blood vessels, reducing vascular permeability, but also enhance perfusion through the remaining normalised vasculature. These opposing effects can thus make the correct interpretation of treatment-induced changes in DCE MRI-derived metrics challenging. This can be compounded in preclinical investigations by the vascular morphology and necrosis that develops in relatively rapidly growing xenograft models, and imperfect compartmental data modelling in rodents.

A number of alternative MRI approaches are thus being exploited to provide simpler yet sensitive imaging biomarkers of tumour angiogenesis and vascular response, and that may provide complementary mechanistic insight when used in conjunction with DCE MRI. One such approach used extensively preclinically is susceptibility contrast MRI using USPIO particles, enabling quantitation of fBV (%), and shown to inform on both incipient tumour angiogenesis (Robinson *et al*, 2008) and response to vascular targeted therapies (Persigehl *et al*, 2007; Robinson *et al*, 2007). In the present study, treatment with vanucizumab significantly decreased tumour fBV *in vivo* compared with omalizumab (control), also aligning with the significantly lower tumour uptake of Hoechst 33342. The susceptibility effects generated by USPIO particles are stronger than gadolinium chelates used in DCE MRI, and thus arguably may provide a more sensitive measurement of tumour response to antivascular/antiangiogenic therapy (Persigehl *et al*, 2007). Although the clinical development of P904 has been halted, recent studies have highlighted the potential off-label use of ferumoxytol as a USPIO MRI contrast agent in the clinic (Bashir *et al*, 2015). Quantitation of tumour fBV, determined using susceptibility contrast MRI with USPIO particles, used in conjunction with DCE MRI, may thus provide a more informative approach to assess changes in human tumour vascular function following treatment within imaging-embedded clinical trials of novel therapies targeting multiple angiogenesis pathways, and to delineate any treatment-induced window of vascular normalisation *in vivo*.

Tumour vascular normalisation has been shown to occur within several days of treatment with a VEGF pathway inhibitor (Winkler *et al*, 2004). The vascular structural features associated with vessel normalisation, including reduced tumour MVD and increased pericyte coverage of the surviving vessels, have been observed following Ang-2 blockade, and in combination studies targeting Ang-2 and VEGF (Falcón *et al*, 2009; Mazzieri *et al*, 2011; Daly *et al*, 2013). Similarly, chronic treatment with vanucizumab has previously been shown to lower MVD while increasing pericyte expression (Kienast *et al*, 2013). In the present study, no such histological evidence of vascular normalisation was identified in Colo205 xenografts following acute single-dose treatment with vanucizumab, a dose regime that may have elicited a more subtle angiogenic response to that reported with chronic dosing (Kienast *et al*, 2013). Our data did demonstrate a significant decrease in MVD of Colo205 xenografts following single-dose treatment with bevacizumab, a similar response to that reported across a number of preclinical tumour models (see, e.g., O'Connor *et al*, 2009; Moestue *et al*, 2013). However, others have demonstrated no such acute effect of bevacizumab on tumour MVD (Yuan *et al*, 1996; Turley *et al*, 2012). The dose scheduling for bevacizumab appears to be important, as chronic dosing has been shown to reduce MVD whereas single administration demonstrated no effect (Yuan *et al*, 1996). In the absence of VEGF, Ang-2 expression can lead to vessel regression (Maisonpierre *et al*, 1997; Lobov *et al*, 2002). Furthermore, targeting Ang-2 in Colo205 xenografts is insufficient to alter MVD (Hashizume *et al*, 2010). It is clear that when considering the highly complex network of angiogenic pathways, the effect on vessel development when targeting single or multiple factors is context, dose and/or time dependent.

During antiangiogenic therapy, dynamic changes in vessel perfusion often result in modifications to tumour oxygenation. Herein, treatment-induced reductions in vessel perfusion (significant with vanucizumab and bevacizumab), as measured by Hoechst 33342 uptake, were associated with a nonsignificant increase in hypoxia in all treatment groups compared with control. Previous work has shown that vanucizumab causes a transient increase in expression of the hypoxia-regulated protein carbonic anhydrase 9 (CAIX) after 10 days of treatment in Colo205 xenografts. This upregulation, presumed to be due to increased hypoxia, may precede and then drive the phenotypic changes associated with vessel normalisation that were observed at later time points (Kienast *et al*, 2013). Similarly, Ang-2 blockade has also been shown to increase tumour hypoxia after a 2-week dosing schedule (Mazzieri *et al*, 2011). Our results suggest that after 5 days of treatment, decreased perfusion may be influencing early changes in tumour oxygenation that in turn may contribute to the observed antitumour activity.

Gene expression profiling of tumour tissues led to the identification of 45 human genes that were differentially expressed after vanucizumab treatment, but were not significantly changed by bevacizumab in this study, thereby representing genes that could explain different treatment effects induced by the two antibodies. Differential expression of 20 genes exclusively in the vanucizumab-treated tumours appeared to be regulated by the combined effect of Ang-2 and VEGF-A inhibition. The expression of 25 other genes was also affected by treatment with LC06, potentially indicating that this regulation is driven by the LC06 component of vanucizumab. Many of the genes are known to be involved in angiogenesis, blood vessel development and/or endothelial cell movement. Other genes have been implicated in microtubule rearrangements or other extracellular processes. Several genes had been previously described as being VEGF-A dependent (Brauer *et al*, 2013), but were not identified in the bevacizumab-treated tumours herein. One explanation for this discrepancy is that Brauer *et al* (2013) used B20-4.1.1 (anti-VEGFA) that is crossreactive with both human and mouse VEGF-A, whereas bevacizumab only targets human VEGF-A. The present results also suggest that these genes are not specifically regulated by bevacizumab, but can also be affected by other antiangiogenic treatments such as anti-Ang2 (LC06), or combined human Ang-2 and VEGF-A inhibition by vanucizumab. Additional tumour models should be profiled to verify the involvement of these genes in the specific mechanism of action of vanucizumab.

In conclusion, using multiparametric MRI combined with *ex vivo* histology, we have demonstrated that treatment of Colo205 xenografts with vanucizumab results in significant antitumour activity associated with a significant reduction in functional tumour vasculature. Gene expression profiling of tumour tissues identified 20 genes that could be specifically attributed to treatment effects of vanucizumab. Targeting both Ang-2 and VEGF with vanucizumab represents a potent antiangiogenic treatment strategy and whose antivascular effects can be monitored *in vivo* using DCE MRI. Incorporation of susceptibility contrast MRI measurements into clinical imaging protocols may provide additional confirmatory evidence of antiangiogenic response to vanucizumab in human tumours.

## Figures and Tables

**Table 1:** Summary of the quantitative DW MRI data

	**ADC (× 10**^**6**^ **mm**^**2**^** s**^**−1**^)	
	**Pre**	**Post**
Vanucizumab	729±24	750±87
Bevacizumab	696±27	786±69
LC06	743±22	734±25
Omalizumab	733±13	687±34

Abbreviations: ADC=apparent diffusion coefficient; DW MRI=diffusion-weighted magnetic resonance imaging.

Estimates of ADC determined from Colo205 xenografts before and 5 days after treatment with either vanucizumab, bevacizumab, LC06 or omalizumab. Data are mean±1 s.e.m., *n*⩾6 per treatment.

## Notes

Markus Thomas, Astrid Koehler, Tapan Nayak, Jean Tessier, Chia-Huey Ooi, Fabian Birzele, Anton Belousov, Magdalena Zajac, Carsten Horn and Clare LeFave were employees of the Roche Group at the time of the study.
