# Supplementary material for: Acute tumour response to a bispecific Ang-2-VEGF-A antibody: insights from multiparametric MRI and gene expression profiling
Source: Br J Cancer. 2016 Aug 16;115(6):691–702. doi: 10.1038/bjc.2016.236 (PMC5023775; doi:10.1038/bjc.2016.236)
Supplement: Supplementary Figure Legend [file bjc2016236x3.docx]

***Supplementary Figure 1 – Differential expression of genes that are regulated by vanucizumab but were not modulated by bevacizumab.*** (a) 24 mouse Ensembl genes (of which 20 are mapped to human gene symbols) showed differential gene expression only in the vanucizumab treated tumour samples. (b) The expression of 25 other genes was affected by LC06 and vanucizumab treatment, but not by bevacizumab. The genes have been mapped to their involvement in a particular biological process and compared to the previously described VEGF-dependent vasculature signature ([Brauer *et al*, 2013](#_ENREF_8)). The differential gene expression between vanucizumab-treated and omalizumab (control) treated tumours are provided as fold-change for experiment 1 (LR(log2 ratio)C1) and 2 (LRC2).
